# Drug-Induced Psychosis: Causes, Symptoms, and Treatment

**DOI:** 10.1192/j.eurpsy.2023.1415

**Published:** 2023-07-19

**Authors:** T. Jupe, E. Myslimi, I. Giannopoulos, B. Zenelaj

**Affiliations:** 1 Psychiatric Hospital of Attica, Athens, Greece; 2 Freelancer Psychiatrist; 3 National Center for Children Treatment and Rehabilitation, Tirane, Albania

## Abstract

**Introduction:**

A relationship between drug abuse and the onset of psychotic symptoms is strongly supported. A struggling clinical dilemma is how to clearly identify a substance-induced psychosis from a primary psychotic illness or a psychotic illness with comorbid substance use.

**Objectives:**

In this review, the presence of associated psychotic symptoms and the differences in clinical presentation will be analyzed for each substance.

**Methods:**

Α bibliographical review was performed using the PubMED platform. All relevant articles were found using the keywords: substance-Induced Psychoses, symptoms, treatment

**Results:**

Present review shows a picture of the complex relationship between psychotic symptoms and the use and abuse of illicit drugs. Furthermore, in most cases, chronological criteria are not sufficient to prove a direct causal effect between the substance and psychosis. The subjects who presented psychotic symptoms after substance abuse seemed to have a higher risk of the development of a primary psychotic illness.

**Conclusions:**

Psychosis due to substance abuse is a common issue in clinical practice and the propensity to develop psychosis seems to be associated with the severity of use and dependence.

**Disclosure of Interest:**

None Declared

